# Intercropping of *Euonymus japonicus* with *Photinia × fraseri* Improves Phytoremediation Efficiency in Cd/Cu/Zn Contaminated Field

**DOI:** 10.3390/biology11081133

**Published:** 2022-07-28

**Authors:** Junli Liu, Gaoyang Qiu, Chen Liu, Yicheng Lin, Xiaodong Chen, Hua Li, Qinglin Fu, Bin Guo

**Affiliations:** State Key Laboratory for Managing Biotic and Chemical Threats to the Quality and Safety of Agro-Products, Institute of Environment, Resource, Soil and Fertilizer, Zhejiang Academy of Agricultural Sciences, Hangzhou 310021, China; liujunli@zaas.ac.cn (J.L.); qiugy@zaas.ac.cn (G.Q.); liuchen@zaas.ac.cn (C.L.); linyc@zaas.ac.cn (Y.L.); chenxiaodong@zaas.ac.cn (X.C.); lihua@zaas.ac.cn (H.L.); fuql@zaas.ac.cn (Q.F.)

**Keywords:** greening plant, heavy metal, intercropping, phytoremediation efficiency

## Abstract

**Simple Summary:**

Intercropping of different greening plant species is a regular management practice in landscape engineering. However, how this approach works on the remediation of heavy metal soil is not fully known. Here, the effect of intercropping two greening species, *Euonymus japonicus* and *Photinia × fraseri* on phytoremediation efficiency was studied in a Cd/Cu/Zn-contaminated field. This study provides (1) a feasible biotechnique for improving phytoremediation efficiency using greening plants and (2) a more practical work for multiple HM-polluted soil than research on single-polluted soil.

**Abstract:**

Intercropping plants for phytoremediation is a promising strategy in heavy metal-polluted soils. In this study, two typical greening plant species, *Euonymus japonicus* (*E. japonicus*) and *Photinia × fraseri* (*P. × fraseri*), were intercropped in a Cd/Cu/Zn-contaminated field. The phytoremediation efficiency was investigated by measuring the plant biomass, metal concentration, and mycorrhizal colonisation, as well as the effects on soil properties, including soil pH; soil total N; and available N, P, K, Cd, Cu, and Zn. The results showed that, compared with the monoculture system, intercropping significantly lowered the available Cd, Cu, and Zn contents, significantly improved the total and available N contents in rhizosphere soils of both plant species, and increased the hyphae colonisation rate of *P. × fraseri*. In both plants, intercropping significantly improved the total plant biomass. Furthermore, the concentrations Zn and Cd in the root of *E. japonicus* and Cu concentration in the root of *P. × fraseri* were enhanced by 58.16%, 107.74%, and 20.57%, respectively. Intercropping resulted in plants accumulating higher amounts of Cd, Cu, and Zn. This was particularly evident in the total amount of Cd in *E. japonicus*, which was 2.2 times greater than that in the monoculture system. Therefore, this study provides a feasible technique for improving phytoremediation efficiency using greening plants.

## 1. Introduction

With the rapid expansion of nonferrous metal mining and smelting activities in China, agricultural fields across the country have been widely contaminated by heavy metals (HMs), which has resulted in a significant risk to the public health. Derived from parent rocks, HMs can be released during the leaching and heating of nonferrous metallic ores and irrigation with the surface water impacted by mining discharges. In addition, the atmospheric deposition of metal-containing dust also impacts agricultural fields located far away, which have sprouted across the regions rich in nonferrous metal resources of China, primarily in Hunan, Guangxi, Guangdong, and Jiangxi Provinces [1]. As reported by the Chinese National Soil Pollution Investigation Bulletin (2014) [2], approximately 19.4% of agricultural land is polluted by HMs, and multi-polluted soil comprises a very high proportion. Generally, multiple metal-combined stresses were more toxic to plants than a single metal stress [3,4,5]. Possible co-toxic mechanisms include the aggravation of oxidative stress [6,7], alteration of plant water [6], mineral nutrition [8], and hormonal status [9], resulting in greater growth reductions compared to a single metal stress [8,10]. However, compared to the research on single metal pollution, fewer studies have been conducted to investigate the phytoremediation techniques for multiple HM-polluted soil.

Intercropping is regarded as an effective technique for enhancing the phytoremediation efficiency [11,12]. For instance, intercropping *Pteris vittata* L. with *Morus alba* L. or *Castanea mollissima* Bl. with *Camellia sinensis* L. significantly enhanced the metal accumulation capacity of both plants by increasing the biomass and metal concentration compared to a monoculture system [13,14]. Intercropping may alter the mutual rhizosphere environments of both plants by accelerating the root exudation [15], improving the metal bioavailability [15], and enhancing the soil enzyme activity and microbial diversity [16,17], among others. Intercropping also strengthens the metal tolerance of plants by increasing the photosynthesis and oxidation resistance [16,17]. Many studies have introduced intercropping systems with hyperaccumulators to remediate contaminated soils. However, disposal of the polluted plant materials remains unfeasible, thereby limiting the use of this technique. Greening plants have been recommended as ideal low-cost candidates for phytoextraction owing to their economical use of follow-up materials [18,19,20]. The intercropping of different greening plant species is a regular management practice in landscape engineering. However, to the best of our knowledge, no studies investigating the effect of intercropping greening plants on metal removal in HM-combined polluted soil have been conducted to date. This pattern can be utilized in polluted agricultural lands, especially for the greening business, which has been occupied a large area of fertile land for marketing.

Arbuscular mycorrhizal fungi (AMF) are beneficial fungi belonging to the phylum Mucoromycota and subphylum Glomeromycotina, which can establish obligately symbiotic association by interacting with about 80% of terrestrial plants [21,22].AMF have beneficial effects on the host plants adapting to heavy metal stress [23]. They interfere with many plant metabolisms, mainly through metal immobilization, nutrient balance, and the activation of transporters involved in HM uptake and transport [24]. Although AM symbioses have been found in most terrestrial plant species, different AM symbioses showed a wide range of plasticity in metal assimilation, reaching from preventing metal transference on the one hand to the highlighting metal accumulation on the other hand. For example, the inoculation of AMF significantly restricted As uptake and retained more As in roots by upregulating *MsPT4* and *MsMT2* in *Medicago sativa* L. [25]. In contrast, the inoculation of AMF led to higher Cd accumulation in *Solanum nigrum* L. by regulating the metal transporters (*Nramp5* and *HMA3*) in the intercropping system with rice and *S. nigrum* [26]. Furthermore, many studies have revealed that the intercropping system increased or decreased AMF diversity by regulating the rhizosphere environment and root distribution [27,28,29]. To date, the contribution of AMF to intercropping greening plants for phytoremediation is still unclear.

*Euonymus japonicus* Thunb. (*E. japonicus*) and *Photinia × fraseri* Dress. (*P. × fraseri*) are very popular ornamental plant for parks and gardens in China. *E. japonicus* has variegated or yellow leaves [30], while *P. × fraseri* has very striking foliage with new bright red leaves and dark green older ones [31]. Furthermore, both of them have bright branches and vast root systems, which can grow well in infertile and contaminated soil [19,32]. Due to the high economic and ecological value, they have been widely applied to landscapes in China. In this study, these two greening plant species were planted in monoculture and intercropping systems to explore the changes in the rhizosphere environment and heavy metal accumulation in Cd/Cu/Zn-polluted soils.

We hypothesised that the soil characteristics and AMF would be affected by intercropping systems in a manner that improved the phytoremediation efficiency. The aims of this study were to evaluate the heavy metal remediation efficiency of the intercropping system and study the changes in the rhizosphere environment and AMF colonisation, as well as their subsequent roles in the phytoremediation of metal-contaminated soil.

## 2. Materials and Methods

### 2.1. Soil and Site Description

The field experiment was performed in paddy soils at a mining site in Zhejiang Province, China (Latitude 30.08219°, Longitude 119.046672°). The heavy metal concentrations and major chemical characteristics of the soil are presented in Table 1. *E. japonicus* and *P. × fraseri* seedlings were collected from the Zhejiang Senhe Seedling Company. The experimental design of the field plot experiment was completely randomised with four replicates and three different cultivation systems: (1) *E. japonicus*, (2) *P. × fraseri*, and (3) *E. japonicus* intercropped with *P. × fraseri*. The row space of *E. japonicus* and *P. × fraseri* was set as 0.5 × 0.4 m, and the planting plot was 8 m^2^ (60 plants in total of every plot; Figure S1). Healthy and uniform plants (1 year old) were planted in the designated field plots. Plants were watered and weeds were removed according to the routine management practices of the local farmers. After 340 d of growth, the plants were harvested, and the planted soil was sampled for further analysis.

### 2.2. Plant and soil Cd, Cu, and Zn Concentration

The plant roots were washed with deionised water; The rhizospheric soil (the cohesive soil from the plant roots) was brushed from the roots, mixed, air-dried, and sieved by 1 mm for further analysis. Leaves, stems, and roots were separated; dried at 70 °C to a constant weight; ground into a powder; passed through the sieves (2 mm); and stored at room temperature before analysis. The sample was subsequently digested in HNO_3_ at 160 °C for 12 h prior to analysis. The soil samples were digested with a mixture of water/HCl/HNO_3_ (1:1:4, *v*/*v*) in a microwave oven (MARS-240, USA CEM, North Carolina, USA) [18]. The concentrations of Cd, Cu, and Zn were measured using inductively coupled plasma mass spectrometry (ICP-MS; 7700, Agilent, Palo Alto, CA, USA).

### 2.3. Soil Extractable Cd, Cu, Zn; Dissolved Organic Carbon; and pH

Cd, Cu, and Zn were extracted using the Community Bureau of Reference (BCR) sequential extraction method [34]. The soil samples were treated with a 0.11-M acetic acid solution and then analysed using ICP-MS (ICP-MS, 7700, Agilent, Palo Alto, USA). The dissolved organic carbon (DOC) was extracted with distilled water at a solid-to-water ratio of 1:2.5 (*w*/*v*) and then shaken at 200 rpm for 2 h at 20 °C. The suspension was filtered through a 0.45-μm membrane filter and then measured using a carbon analyser (Multi N/C 3100, Analytikjena, Jena, Germany) [35]. The soil organic matter was determined using the dichromate wet oxidation method [36]. The soil pH was determined according to the ratio of soil to water 1:2.5 (*w*/*v*) for dissolution and subsequently examined using a pH meter (PHS-2F, Leici, Shanghai, China)

### 2.4. Detection of the Root Mycorrhization Rate

The plant roots were washed with water and separated randomly into segments (1 to 2 cm). They were then treated with 10% KOH for 1.2 h at 90 °C, washed with water and acidified with 5% (*v*/*v*) HCl solution for 8 min at 25 °C, and stained with 0.2% (*w*/*v*) trypan blue solution at 90 °C for 2 h [37]. The root segments were analysed for mycorrhizal colonisation using the magnified line intersection method (OLYMPUS DP74, Tokyo, Japan) [38].

### 2.5. Calculation of Bioconcentration Factor, Land Equivalent Ratio, and Metal Removal Equivalent Ratio

The plant bioconcentration factor (BCF) is commonly used to assess the potential of plants to remove heavy metals and was calculated using the following formulas [39]:

BCF_R_ = (HM concentration of root)/(HM concentration in soil)

BCF_S_ = (HM concentration of stem)/(HM concentration in soil)

BCF_L_ = (HM concentration of leaf))/(HM concentration in soil)

The land equivalent ratio (LER) is commonly used to assess the intercropping advantage and is calculated as follows [40]:

LER = (Y_A_I/Y_A_M) + (Y_B_I/Y_B_M)

where Y_A_I and Y_A_M indicate the yields of plant A in the intercropping and monoculture systems, respectively. Y_B_I and Y_B_M indicate the yields of plant B in the intercropping and monoculture systems, respectively. LER > 1 or <1 indicates the yield advantage or disadvantage of intercropping, while LER = 1 shows the same resource utilisation efficiency between intercropping and the monoculture.

The metal removal equivalent ratio (MRER) is defined by the following formula [41]:

MRER= (X_A_I/X_A_M) + (X_B_I × X_B_M) (1)

where X_A_I and X_A_M represent the heavy metal content of plant A in the intercropping and monoculture systems, respectively. X_B_I and X_B_M represent the heavy metal content of plant B in the intercropping and monoculture systems, respectively.

### 2.6. Statistical Analysis

The experimental data were calculated using Excel 2013 (shown as the mean ± standard error), followed by statistical analysis using SPSS 20.0. The means were subjected to a test of statistical significance using Duncan’s multiple range test at a 5% probability level [18].

## 3. Results

### 3.1. Plant Biomass

**Figure 1:**
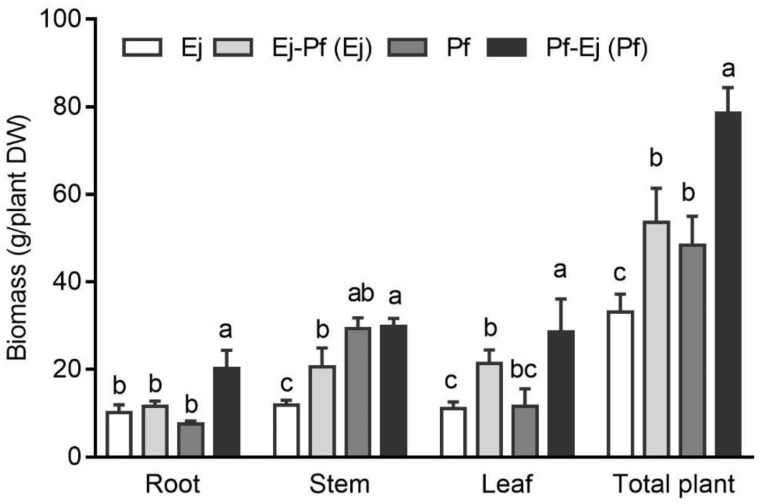
Biomass of *P. × fraseri* and *E. japonicus* in the monoculture and intercropping systems. Ej and Pf refer to *E. japonicus* and *P. × fraseri* in the monoculture system, respectively; Ej-Pf and Pf-Ej (Pf) refer to *E. japonicus* and *P. × fraseri* in the intercropping system, respectively. All values are presented as the mean ± standard error (*n* = 4), and bars with different lowercase letters indicate significant (*p* < 0.05) differences between the treatments and control, according to Duncan’s test.

Plant biomass is an important factor for evaluating the efficiency of phytoremediation. The dry weights of the root, stem, leaf, and total plant were determined over the course of the experimental period (340 d) (Figure 1). *P. × fraseri* had a higher stem (by 146.25%) than *E. japonicus*. Intercropping significantly enhanced the stem and total biomass for *E. japonicus* and roots, leaves, and total biomass for *P. × fraseri*. For example, the stem, leaf, and total plant dry weights of *E. japonicus* increased by 72.71%, 93.85%, and 61.60%, respectively. The improvement in root and leaf dry weights was more pronounced in *P. × fraseri*, with 165.56% and 146.82% increases compared to the monoculture system, respectively. The total biomass of *P. × fraseri* in the intercropping system was 62.21% higher than that for *P. × fraseri* in the monoculture system.

### 3.2. Concentration of Total Cd/Cu/Zn in Plants

**Figure 2:**
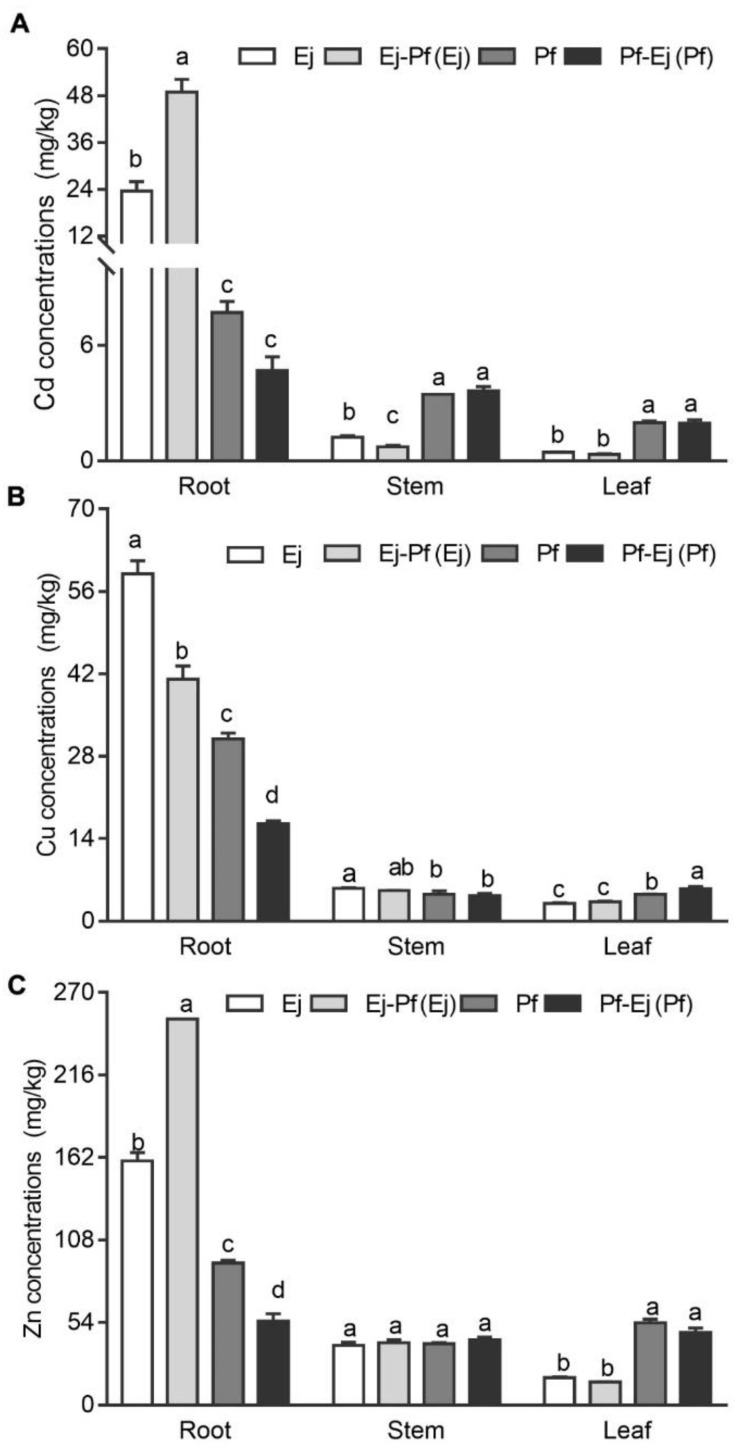
The Cd, Cu, and Zn concentrations of *P. × fraseri* and *E. japonicus* in the monoculture and intercropping systems. Ej and Pf refer to *E. japonicus* and *P. × fraseri* in the monoculture system, respectively; Ej-Pf and Pf-Ej (Pf) refer to *E. japonicus* and *P. × fraseri* in the intercropping system, respectively. The concentrations of Cd (**A**), Cu (**B**), and Zn (**C**) in the root, stem, and leaf for *P. × fraseri* and *E. japonicus*, respectively. All values are presented as the mean ± standard error (*n* = 4), and bars with different lowercase letters indicate significant (*p* < 0.05) differences between the treatments and control, according to Duncan’s test.

As shown in Figure 2, *E. japonicus* accumulated significantly higher (*p* < 0.05) levels of Cu, Zn, and Cd in the roots but lower Cu, Zn, and Cd in the leaves than *P. × fraseri* in the monoculture system over the course of the experimental period. *E. japonicus* also showed less Cd concentration in stems when compared to *P. × fraseri*. Intercropping significantly increased the translocation of Cd (by 107.74%) and Zn (by 58.16%) in roots (*p* < 0.05) (Figure 2A,C) and reduced the translocation of Cd in stems (by 39.20%) and Cu in roots (by 30.42%) *E. japonicus* (*p* < 0.05) (Figure 2A,B), while intercropping decreased the translocation of Cu (by 46.64%) and Zn (by 20.57%) in roots but increased the translocation of Cu in leaves of *P. × fraseri* (*p* < 0.05) (Figure 2B,C).

### 3.3. Total Cd/Cu/Zn content in P. × fraseri and E. japonicus

**Figure 3:**
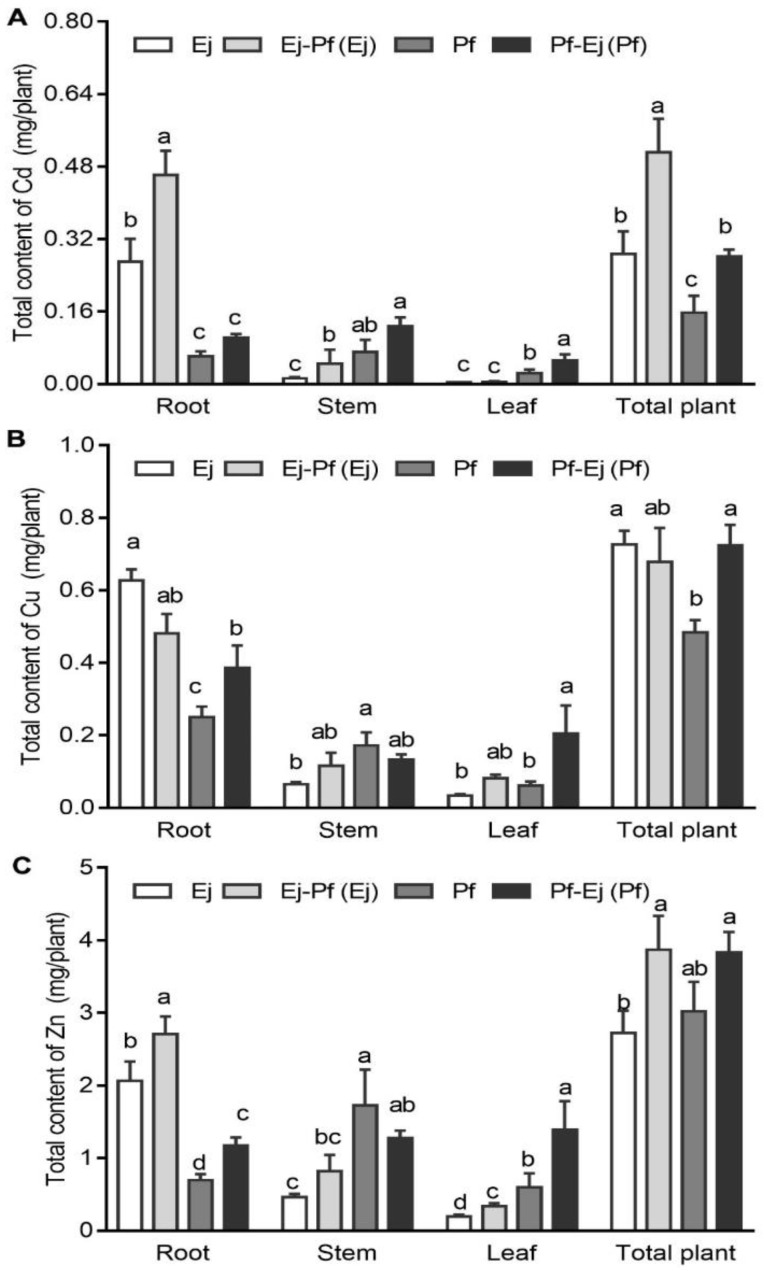
The Cd, Cu, and Zn contents of *P. × fraseri* and *E. japonicus* in the monoculture and intercropping systems. Ej and Pf refer to *E. japonicus* and *P. × fraseri* in the monoculture system, respectively; Ej-Pf and Pf-Ej (Pf) refer to *E. japonicus* and *P. × fraseri* in the intercropping system, respectively. The total contents of Cd (**A**), Cu (**B**), and Zn (**C**) in the root, stem, leaf, and total plant, respectively. All values are presented as the mean ± standard error (*n* = 4), and bars with different lowercase letters indicate significant (*p* < 0.05) differences between the treatments and control, according to Duncan’s test.

In the monoculture system, the distribution of the total metal content was similar to that of the metal concentrations, as noted in that 86.38% Cu, 77.78% Zn, and 94.08% Cd were accumulated in the roots of *E. japonicus*, while 12.92% Cu, 20% Zn, and 12.7% Cd were accumulated in the leaves of *P. × fraseri* (Figure 3). Intercropping significantly altered the accumulation of these metals in both plant species. For example, the total Cu content in the roots and leaves of *P. × fraseri* increased by 53.99% and 232.47%, respectively, whereas that in the roots of *E. japonicus* significantly decreased by 31.37% (*p* < 0.05) (Figure 3B). The total Zn content of the leaves in *P. × fraseri* increased by 130.04% (Figure 3C). The total content of Cd in the stems and leaves of *P. × fraseri* increased by 80.28% and 122.98%, respectively; furthermore, a significant increase was found in the total Cd content in the roots (by 70.61%) and stems (by 244.19%) of *E. japonicus* in the intercropping system when compared with the monoculture (Figure 3A). The total contents of Zn and Cd in the *E. japonicus* whole plant and the total content of Cu in the *P. × fraseri* whole plant were significantly higher (*p* < 0.05) in the intercropping system and increased by 42.07%, 78.13%, and 49.45% (Figure 3), respectively.

### 3.4. BCF, MRER, and LER of P. × fraseri and E. japonicus in the Monoculture and Intercropping Systems

The BCF was calculated as the ratio of the metal concentration in the plant parts to that of the corresponding soil in which the plant was grown. Generally, the BCF of Cd was much higher than those of Zn and Cu (Table 2), indicating that Cd is easily absorbed by plants (Table 2). Intercropping significantly enhanced the BCF of Cd in the roots of *E. japonicus* and the stems and leaves of *P. × fraseri* (*p* < 0.05). A similar effect of intercropping was also noted in the BCF of Zn. Furthermore, the LER was 3.62, indicating that intercropping *E. japonicus* with *P. × fraseri* had a distinct yield advantage. Additionally, the MRERs of Cu, Zn, and Cd in the intercropping system were 2.48, 2.85, and 3.32, respectively, suggesting that the intercropping pattern has an advantage in removing heavy metals, particularly Cd.

### 3.5. Soil Chemical Characteristics and Heavy Metal Concentrations in Monoculture and Intercropping Systems

A comparison of the soil chemical characteristics, including pH; total N; SOM; and available N, P, K, Cu, Zn, and Cd, between the monoculture and intercropping systems is provided in Table 3. For both studied plants, the available Cu, Zn, and Cd of the soil in the intercropping system were significantly lower than in the monoculture system, except for Zn in the rhizosphere soil of *P. × fraseri*. Intercropping also enhanced the N level of the soil, as noted by the increase in the total and available N in *E. japonicus* (47.10% and 29.78%, respectively) and *P. × fraseri* (61.99% and 46.08%, respectively). In addition, the soil pH significantly decreased (*p* < 0.05) in the rhizosphere soil of intercropped *E. japonicus* compared with the monoculture. Furthermore, the available K significantly increased (*p* < 0.05) in the rhizosphere soil of *E. japonicus* and significantly decreased in the rhizosphere soil of *P. × fraseri*.

### 3.6. Mycorrhization Rate of P. × fraseri and E. japonicus in Monoculture and Intercropping Systems

**Figure 4:**
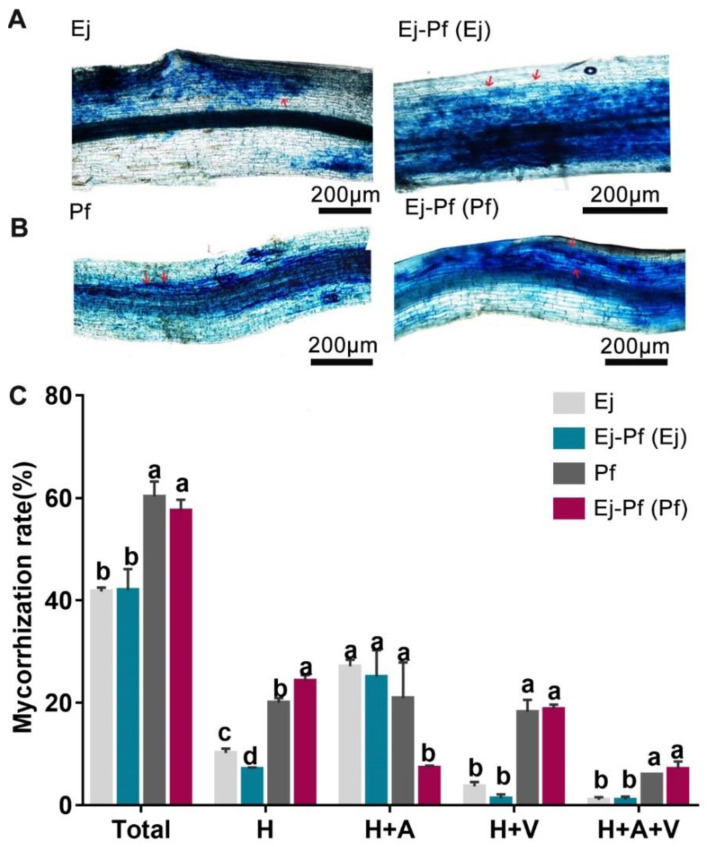
The analysis of the mycorrhization rate of *P. × fraseri* and *E. japonicus* in the monoculture and intercropping systems. (**A**,**B**) Images of *E. japonicus* and P. × fraseri roots, bar = 200 µm. (**C**): Quantification of the mycorrhization rate level of *P. × fraseri* and *E. japonicus* plants. Ej and Pf refer to *E. japonicus* and *P. × fraseri* in the monoculture system, respectively; Ej-Pf and Pf-Ej (Pf) refer to *E. japonicus* and *P. × fraseri* in the intercropping system, respectively. A, H, and V represent “arbuscules”, “hyphae”, and “vesicles”, respectively; A + H (%) indicates the percentage of root with arbuscules and hyphae; A + V + H (%) represents the percentage of root with arbuscules, vesicles, and hyphae; H (%) indicates percentage of root with only hyphae; and V + H (%) indicates the percentage of root with vesicles and hyphae. Red arrows indicate arbuscules. All values are presented as the mean ± standard error (*n* = 4), and bars with different lowercase letters indicate significant (*p* < 0.05) differences between the treatments and control, according to Duncan’s test.

To assess the influence of the intercropping system on the mycorrhization rate and plant performance, the degree of AMF colonisation was calculated (Figure 4). We observed that fungal hyphae and arbuscules were branched in the colonised roots in all the treatments (Figure 4A). In the monoculture system, *P. × fraseri* plants showed a significantly higher colonisation rate in the total, hyphae, and vesicles than *E. japonicus* plants by 44.51%, 96.95%, and 397.27% (*p* < 0.05), respectively (Figure 4B). However, substantially lower colonisation [H + A (%)] (64.88%) was observed in *P. × fraseri* plants compared to the monoculture system, and the proportion of root sectors containing hyphae (H (%)) was significantly higher (*p* < 0.05) in intercropped *P. × fraseri* plants than in monoculture plants by 21.2% (Figure 4B). In summary, the results suggest that mycorrhizal symbiosis was affected in intercropped plants.

## 4. Discussion

Phytoremediation with hyperaccumulators has been a promising technology for agricultural lands. However, the disposition of a hazardous biomass after mediation is still unfeasible. Thus, we perilously proposed a new technique of uptake and stabilizing the HMs from the contaminated soil by greening plants prior to their intravital transplantation for economical landscaping [18]. The pattern of high root retention (as also shown in the Figure 2) and low risk for defoliation (as in evergreen species) contribute to the feasibility of this technique for soil cleansing. More importantly, it is an eco-friendly technique, which would providing a feasible solution to the remediation of agricultural land polluted by HMs. In this study, we set up the intercropping system with different greening species, which aimed to further improve the efficiency of this technique.

### 4.1. Differences in P. × fraseri and E. japonicus Biomass in Monoculture and Intercropping Systems

Greening plants can be utilized for phytoremediation, as they have a marketable biomass compared with classical hyperaccumulators [18]. As observed in this study, the biomass of *P. × fraseri* and *E. japonicus* (48 and 33 g/plant, respectively) (Figure 1) are approximately 11–137 times larger than that of *Sedum alfredii* Hance. [40,42].

Intercropping significantly promoted the growth of both *P. × fraseri* and *E. japonicus*, as indicated by the high LER (Figure 1 and Table 2). Growth stimulation has also been observed in other systems, such as the intercropping of oilseed rape with *Sedum alfredii* [43], *Sonchus asper (L.) Hill* with *Vicia faba* Linn. [44], and *Sedum alfredii* with *Zea mays* Linn. [45]. This might because intercropping positively regulates the nutrient bioavailability through intertwined root exudation [15,46,47]. The root exudates could break the SOM–mineral connection, thus leading to the release of DOC in the soil [48]. As observed in this study, intercropping slightly decreased the SOM content (about 2 g/kg) and correspondingly increased the content of DOC in the Ej-Pf part (Table 1 and Table 3). It was also reported that the application of low-molecular-weight organic acids significantly reduced the SOM by 28.1% after 15 days [49]. Furthermore, the degradation of SOM by intercropping considerably affected the mycorrhization rate of both plant species (Figure 4), which, in turn, enhanced the total and available N of the rhizosphere soils (Table 3). A similar phenomenon was observed in the *Manihot esculenta* Crantz./*Arachis hypogaea* Linn. system [50,51].

The enhancement of growth was more pronounced in *P. × fraseri* compared to *E. japonicus* in the intercropping system. This might because *P. × fraseri* is a taller and broad shrub, which had a much higher biomass than *E. japonicus* (as seen in Figure S1). The fast growth of *P. × fraseri* could facilitate the allocation of more resources from the soil, including space, water, and nutrients. Similar results were also found in a previous study of intercropping red *Solanum melongena* L. (*S. melongena*) with green *S. melongena* and intercropping green *S. melongena* with black *S. melongena*, and rice (*Oryza sativa* Linn.) intercropping with *Solanum nigrum* L. decreased the growth of red eggplant, black *S. melongena*, and rice, respectively [26,52]. The growth discrepancy also changed the soil pH differently, as noted that *P. × fraseri* induced a strong decreasing effect on the soil pH, which even covered the increased effect of *E. japonicus* in the intercropping system (Table 3). Furthermore, AMF colonisation could influence the process of metal uptake by the plant. The total colonisation rate of *P. × fraseri* was higher than that of *E. japonicus* in both the monoculture and intercropping systems (Figure 4), indicating that intercropping affected the soil microbial community of the rhizosphere. It was also reported that Moso bamboo (*Phyllostachys edulis* J. Houz.) intercropping with *Sedum plumbizincicola* enhanced HM remediation through changing the abundances of the rhizospheric microbes [53]. The transcriptional regulation of AMF-induced genes involved in HM accumulation in plants and the nutritional status of soil requires further study.

### 4.2. Effect of Intercropping on Heavy Metal Accumulation and Phytoremediation Potential in P. × fraseri and E. japonicus

Interestingly, the enhanced DOC contents and decreased soil pH by intercropping failed to increase the bioavailability of heavy metals. In contrast, the bioavailable Cu and Cd were significantly decreased compared with the monoculture system (Table 3), which might because a large amount of them was assimilated by the plants. Intercropping significantly decreased the root/shoot ratio of *E. japonicus* while significantly enhancing the root/shoot ratio of *P. × fraseri* (as shown in Figure S2), It also disrupted the metal distribution among different organs, as observed in the increase in root Cd of *E. japonicus* and leaf Cu of *P. × fraseri*, while decreasing in the stem Cd of *E. japonicus* and the root Cu of *P. × fraseri* (Figure 2A,B), respectively. The change in the metal balance in the intercropping systems was also found in fava beans with *Sedum alfredii* [40], *Galium aparine Linn.* with *Malachium* Fr. ex Rchb. [54], and Moso bamboo with *Sedum plumbizincicola* [53].

Although the metal concentration in some organs was not altered or even lowered in the intercropping system, the total Cd/Cu contents in *P. × fraseri* and *E. japonicus* significantly increased. For example, the Cd concentration in the leaves of *P. × fraseri* did not change, whereas the total Cd accumulation in the leaves increased by 123%. The Cu concentration in the roots of *P. × fraseri* and Cd concentration in the stems of *E. japonicus* decreased by 46% and 41.49%, respectively, while the total Cu in roots and Cd content in the stems increased by 53.99% and 54.05%, respectively, in the intercropping system. Similar results have been reported in the *Conyza canadensis*/*Cerastium glomeratum* and *Stellaria media*/*Malachium aquaticum* systems [54,55]. These results indicate that phytoremediation efficiency depends not only on the metal concentration but also on the biomass of plants.

## 5. Conclusions

In this study, we demonstrated that intercropping with greening plants could promote heavy metal phytoextraction. In the intercropping system, *E. japonicus* showed a higher Cd phytoextraction efficiency than *P. × fraseri*, which was mainly ascribed to the high increase in the plant biomass. Additionally, the concentrations of the soil nutrients were greatly improved, especially the total and available N of the rhizosphere soils (Table 3), which supports the feasibility of this phytoremediation technique. Further studies are necessary to determine whether aspects such as optimal fertilisation and the application of microbial and mobilising agents may contribute to implementation.

## Figures and Tables

**Table 1:** The soil chemical characteristics and heavy metal concentrations in the soil.

Chemical Characteristics	Value	Heavy Metal Concentration	Value	* Standard
pH	4.97	Cu (mg/kg)	97.80	50
Total N (g/kg)	4.56	Zn (mg/kg)	305.89	200
SOM (g/kg)	28.37	Cd (mg/kg)	4.80	0.3
DOC (mg/kg)	144.12	BCR-Cu (mg/kg)	10.23	
Available N (mg/kg)	160.94	BCR-Zn (mg/kg)	16.47	
Available P (mg/kg)	2.65	BCR-Cd (mg/kg)	2.08	
Available K (mg/kg)	48.07			

BCR-Cu, BCR-Zn, and BCR-Cd indicated Cu, Zn, and Cd extracted by the BCR sequential extraction method, respectively, which were generally considered as readily bioavailable factions in the soil [33]. DOC and SOM indicated dissolved organic carbon and soil organic matter, respectively. * Soil environmental quality control standard for the soil pollution risk of agricultural land, 2018, China.

**Table 2:** The BCF, MRER, and LER of *P. × fraseri* and *E. japonicus* in different planting patterns.

Organs	Treatments	Cu	Zn	Cd
BCF_R_	Ej	0.62 ± 0.07 a	0.57 ± 0.05 b	5.54 ± 1.21 b
	Ej-Pf (Ej)	0.68 ± 0.05 a	0.81 ± 0.09 a	11.77 ± 1.56 a
	Pf	0.37 ± 0.02 b	0.29 ± 0.01 c	1.45 ± 0.08 c
	Ej-Pf (Pf)	0.27 ± 0.01 b	0.19 ± 0.02 c	1.24 ± 0.08 c
BCF_S_	Ej	0.06 ± 0.00 b	0.13 ± 0.01 ab	0.30 ± 0.04 b
	Ej-Pf (Ej)	0.08 ± 0.00 a	0.14 ± 0.00 ab	0.21 ± 0.02 b
	Pf	0.05 ± 0.01 b	0.12 ± 0.00 b	0.42 ± 0.15 b
	Ej-Pf (Pf)	0.06 ± 0.00 b	0.15 ± 0.01 a	0.96 ± 0.07 a
BCF_L_	Ej	0.03 ± 0.00 c	0.06 ± 0.00 b	0.10 ± 0.00 c
	Ej-Pf (Ej)	0.05 ± 0.00 b	0.05 ± 0.00 b	0.09 ± 0.01 c
	Pf	0.05 ± 0.00 b	0.18 ± 0.01 a	0.39 ± 0.02 b
	Ej-Pf (Pf)	0.08 ± 0.01 a	0.17 ± 0.01 a	0.46 ± 0.02 a
MRER	Ej-Pf	2.48	2.85	3.32
LER	Ej-Pf	3.26		

Ej and Pf refer to *E. japonicus* and *P. × fraseri* in the monoculture system, respectively; Ej-Pf and Pf-Ej (Pf) refer to *E. japonicus* and *P. × fraseri* in the intercropping system, respectively. BCF_R,_ BCF_S_, and BCF_L_ indicate the BCF of the root, stem, and leaf, respectively. All values are presented as the mean ± standard error (*n* = 4), and bars with different lowercase letters indicate significant (*p* < 0.05) differences between the treatments and control, according to Duncan’s test.

**Table 3:** The soil chemical characteristics and heavy metal concentrations in the rhizosphere soil of *P. × fraseri* and *E. japonicus* in different planting patterns.

Treatments		Ej	Ej-Pf (Ej)	Pf	Ej-Pf (Pf)
The chemical characteristics of Soil	pH	5.21 ± 0.04 a	4.87 ± 0.03 b	4.82 ± 0.01 b	4.84 ± 0.05 b
	Total N (g/kg)	6.37 ± 0.13 b	9.37 ± 0.22 a	6.75 ± 0.22 b	8.76 ± 0.38 a
	SOM (g/kg)	27.81 ± 0.68 ab	25.67 ± 0.51 c	28.80 ± 0.81 a	26.07 ± 0.51 bc
	TOC (mg/kg)	127.25 ± 10.33 a	151.35 ± 5.67 a	141.31 ± 12.27 a	141.98 ± 3.42 a
	Available N (mg/kg)	141.40 ± 2.84 b	229.05 ± 2.74 a	153.31 ± 8.98 b	223.96 ± 3.66 a
	Available P (mg/kg)	2.40 ± 0.06 b	3.10 ± 0.08 a	3.10 ± 0.13 a	2.54 ± 0.12 b
	Available K (mg/kg)	39.37 ± 1.69 b	36.51 ± 1.96 b	51.64 ± 0.69 a	35.05 ± 0.93 b
Available heavy metal concentrations	Cu (mg/kg)	8.06 ± 0.58 a	5.06 ± 0.08 b	8.75 ± 0.98 a	5.42 ± 0.10 b
	Zn (mg/kg)	16.22 ± 0.36 a	14.38 ± 0.40 b	16.47 ± 0.56 a	15.35 ± 0.73 ab
	Cd (mg/kg)	1.97 ± 0.12 a	1.39 ± 0.03 b	2.13 ± 0.04 a	1.62 ± 0.05 b

Ej and Pf refer to *E. japonicus* and *P. × fraseri* in the monoculture system, respectively; Ej-Pf and Pf-Ej (Pf) refer to *E. japonicus* and *P. × fraseri* in the intercropping system, respectively. DOC and SOM indicate dissolved organic carbon and soil organic matter, respectively. All values are represented as the mean ± standard error (*n* = 4), and bars with different lowercase letters indicate significant (*p* < 0.05) differences between the treatments and control, according to Duncan’s test.

## Data Availability

Not applicable.

## Supplementary Materials

The following supporting information can be downloaded at: https://www.mdpi.com/article/10.3390/biology11081133/s1, Figure S1: Picture and schematic diagram of field experiment; Figure S2: The analysis of root/shoot ratio of *P. × fraseri* and *E. japonicus* in monoculture and intercropping systems.

## Abbreviations

AMF	Arbuscular mycorrhiza fungi
BCR	Community Bureau of Reference
BCF	Calculation of bioconcentration factor
DOC	Dissolved organic carbon
Ej/*E. japonicus*	*Euonymus japonicus*
HMs	Heavy metals
LER	Land equivalent ratio
MRER	Metal removal equivalent ratio
Pf/*P. × fraseri*	*Photinia × fraseri* Dress
SOM	Soil organic matter

## References

[1] Hu Y., Cheng H., Tao S. (2016). The challenges and solutions for cadmium-contaminated rice in China: A critical review. Environ. Int..

[2] Ministry of Environmental Protection (2014). A National Survey of Soil Pollution Bulletin.

[3] Sasmaz M., Öbek E., Sasmaz A. (2019). Bioaccumulation of cadmium and thallium in Pb-Zn tailing waste water by *Lemna minor* and *Lemna gibba*. Appl. Geochem..

[4] Chen W., Peng L., Hu K., Zhang Z., Peng C., Teng C., Zhou K. (2020). Spectroscopic response of soil organic matter in mining area to Pb/Cd heavy metal interaction: A mirror of coherent structural variation. J. Hazard. Mater..

[5] Bamagoos A.A., Alharby H.F., Abbas G. (2022). Differential Uptake and Translocation of Cadmium and Lead by Quinoa: A Multivariate Comparison of Physiological and Oxidative Stress Responses. Toxics.

[6] Srivastava R.K., Pandey P., Rajpoot R., Rani A., Dubey R. (2014). Cadmium and lead interactive effects on oxidative stress and antioxidative responses in rice seedlings. Protoplasma.

[7] Kaya C., Akram N.A., Sürücü A., Ashraf M. (2019). Alleviating effect of nitric oxide on oxidative stress and antioxidant defence system in pepper (*Capsicum annuum* L.) plants exposed to cadmium and lead toxicity applied separately or in combination. Sci. Hortic..

[8] He H., Wang X., Wu M., Guo L., Fan C., Peng Q. (2020). Cadmium and lead affect the status of mineral nutrients in alfalfa grown on a calcareous soil. Soil Sci. Plant Nutr..

[9] Zhou M., Han R., Ghnaya T., Lutts S. (2018). Salinity influences the interactive effects of cadmium and zinc on ethylene and polyamine synthesis in the halophyte plant species *Kosteletzkya pentacarpos*. Chemosphere.

[10] Ronzan M., Piacentini D., Fattorini L., Della Rovere F., Eiche E., Riemann M., Altamura M., Falasca G. (2018). Cadmium and arsenic affect root development in *Oryza sativa* L. negatively interacting with auxin. Environ. Exp. Bot..

[11] De Conti L., Ceretta C.A., Melo G.W., Tiecher T.L., Silva L.O., Garlet L.P., Mimmo T., Cesco S., Brunetto G. (2019). Intercropping of young grapevines with native grasses for phytoremediation of Cu-contaminated soils. Chemosphere.

[12] Niu H., Wang Z., Song J., Long A., Cao M., Luo J. (2021). Cadmium subcellular distribution and chemical form in *Festuca arundinacea* in different intercropping systems during phytoremediation. Chemosphere.

[13] Ma Y.-h., Fu S.-l., Zhang X.-p., Zhao K., Chen H.Y. (2017). Intercropping improves soil nutrient availability, soil enzyme activity and tea quantity and quality. Appl. Soil Ecol..

[14] Zeng P., Guo Z., Xiao X., Peng C., Feng W., Xin L., Xu Z. (2019). Phytoextraction potential of *Pteris vittata* L. co-planted with woody species for As, Cd, Pb and Zn in contaminated soil. Sci. Total Environ..

[15] Li Z.-R., Wang J.-X., An L.-Z., Tan J.-B., Zhan F.-D., Wu J., Zu Y.-Q. (2019). Effect of root exudates of intercropping *Vicia faba* and *Arabis alpina* on accumulation and sub-cellular distribution of lead and cadmium. Int. J. Phytoremediation.

[16] Gong X., Liu C., Li J., Luo Y., Yang Q., Zhang W., Yang P., Feng B. (2019). Responses of rhizosphere soil properties, enzyme activities and microbial diversity to intercropping patterns on the Loess Plateau of China. Soil Tillage Res..

[17] Zeng P., Guo Z., Xiao X., Peng C. (2019). Dynamic response of enzymatic activity and microbial community structure in metal (loid)-contaminated soil with tree-herb intercropping. Geoderma.

[18] Guo B., Liang Y., Fu Q., Ding N., Liu C., Lin Y., Li H., Li N. (2012). Cadmium stabilization with nursery stocks through transplantation: A new approach to phytoremediation. J. Hazard. Mater..

[19] Zhang T., Bai Y., Hong X., Sun L., Liu Y. (2017). Particulate matter and heavy metal deposition on the leaves of *Euonymus japonicus* during the East Asian monsoon in Beijing, China. PLoS ONE.

[20] Mattei P., Gnesini A., Gonnelli C., Marraccini C., Masciandaro G., Macci C., Doni S., Iannelli R., Lucchetti S., Nicese F.P. (2018). Phytoremediated marine sediments as suitable peat-free growing media for production of red robin photinia (*Photinia* x *fraseri*). Chemosphere.

[21] Smith S.E., Read D.J. (2010). Mycorrhizal Symbiosis.

[22] Spatafora J.W., Chang Y., Benny G.L., Lazarus K., Smith M.E., Berbee M.L., Bonito G., Corradi N., Grigoriev I., Gryganskyi A. (2016). A phylum-level phylogenetic classification of zygomycete fungi based on genome-scale data. Mycologia.

[23] Errisson I., Hognason J., Vidarsdottir H., Gottskalksson G., Gunnarsson G., Sverrisson J., Gudbjartsson T., Dhalaria R., Kumar D., Kumar H. (2020). Arbuscular Mycorrhizal Fungi as Potential Agents in Ameliorating Heavy Metal Stress in Plants. Agronomy.

[24] Shi W., Zhang Y., Chen S., Polle A., Rennenberg H., Luo Z.B. (2019). Physiological and molecular mechanisms of heavy metal accumulation in nonmycorrhizal versus mycorrhizal plants. Plant Cell Environ..

[25] Li J., Sun Y., Jiang X., Chen B., Zhang X. (2018). Arbuscular mycorrhizal fungi alleviate arsenic toxicity to *Medicago sativa* by influencing arsenic speciation and partitioning. Ecotoxicol. Environ. Saf..

[26] Yang X., Qin J., Li J., Lai Z., Li H. (2021). Upland rice intercropping with *Solanum nigrum* inoculated with arbuscular mycorrhizal fungi reduces grain Cd while promoting phytoremediation of Cd-contaminated soil. J. Hazard. Mater..

[27] Lian T., Mu Y., Ma Q., Cheng Y., Gao R., Cai Z., Jiang B., Nian H. (2018). Use of sugarcane–soybean intercropping in acid soil impacts the structure of the soil fungal community. Sci. Rep..

[28] Mei X., Wang Y., Li Z., Larousse M., Pere A., da Rocha M., Zhan F., He Y., Pu L., Panabières F. (2022). Root-associated microbiota drive phytoremediation strategies to lead of *Sonchus Asper* (L.) Hill as revealed by intercropping-induced modifications of the rhizosphere microbiome. Environ. Sci. Pollut. Res..

[29] Zhang R., Mu Y., Li X., Li S., Sang P., Wang X., Wu H., Xu N. (2020). Response of the arbuscular mycorrhizal fungi diversity and community in maize and soybean rhizosphere soil and roots to intercropping systems with different nitrogen application rates. Sci. Total Environ..

[30] Ruiz-Riaguas A., Fernández-de Córdova M., Llorent-Martínez E.J. (2020). Phenolic profile and antioxidant activity of *Euonymus japonicus* Thunb. Nat. Prod. Res..

[31] Larraburu E.E., Apóstolo N.M., Llorente B.E. (2010). Anatomy and morphology of photinia (*Photinia* × *fraseri* Dress) in vitro plants inoculated with rhizobacteria. Trees.

[32] Lin X., Shu D., Zhang J., Chen J., Zhou Y., Chen C. (2021). Dynamics of particle retention and physiology in *Euonymus japonicus* Thunb. var. aurea-marginatus Hort. with severe exhaust exposure under continuous drought. Environ. Pollut..

[33] Guo B., Hong C., Tong W., Xu M., Huang C., Yin H., Lin Y., Fu Q. (2020). Health risk assessment of heavy metal pollution in a soil-rice system: A case study in the Jin-Qu Basin of China. Sci. Rep..

[34] Kartal Ş., Aydın Z., Tokalıoğlu Ş. (2006). Fractionation of metals in street sediment samples by using the BCR sequential extraction procedure and multivariate statistical elucidation of the data. J. Hazard. Mater..

[35] Qu X., Fu H., Mao J., Ran Y., Zhang D., Zhu D. (2016). Chemical and structural properties of dissolved black carbon released from biochars. Carbon.

[36] Nelson D.W., Sommers L.E., Sparks D.L., Page A.L., Helmke P.A., Loeppert R.H., Soltanpour P.N., Tabatabai M.A., Johnston C.T., Sumner M.E. (1996). Total carbon, organic carbon, and organic matter. Methods of Soil Analysis: Part 3 Chemical Methods.

[37] Liu J., Liu J., Chen A., Ji M., Chen J., Yang X., Gu M., Qu H., Xu G. (2016). Analysis of tomato plasma membrane H+-ATPase gene family suggests a mycorrhiza-mediated regulatory mechanism conserved in diverse plant species. Mycorrhiza.

[38] Xue L., Klinnawee L., Zhou Y., Saridis G., Vijayakumar V., Brands M., Dörmann P., Gigolashvili T., Turck F., Bucher M. (2018). AP2 transcription factor CBX1 with a specific function in symbiotic exchange of nutrients in mycorrhizal *Lotus japonicus*. Proc. Natl. Acad. Sci. USA.

[39] Jamali M.K., Kazi T.G., Arain M.B., Afridi H.I., Jalbani N., Kandhro G.A., Shah A.Q., Baig J.A. (2009). Heavy metal accumulation in different varieties of wheat (*Triticum aestivum* L.) grown in soil amended with domestic sewage sludge. J. Hazard. Mater..

[40] Tang L., Hamid Y., Zehra A., Sahito Z.A., He Z., Beri W.T., Khan M.B., Yang X. (2020). Fava bean intercropping with *Sedum alfredii* inoculated with endophytes enhances phytoremediation of cadmium and lead co-contaminated field. Environ. Pollut..

[41] Zeng L., Lin X., Zhou F., Qin J., Li H. (2019). Biochar and crushed straw additions affect cadmium absorption in cassava-peanut intercropping system. Ecotoxicol. Environ. Saf..

[42] Feng Y., Wang Q., Meng Q., Liu Y., Pan F., Luo S., Wu Y., Ma L., Yang X. (2019). Chromosome doubling of *Sedum alfredii* Hance: A novel approach for improving phytoremediation efficiency. J. Environ. Sci..

[43] Cao X., Wang X., Lu M., Hamid Y., Lin Q., Liu X., Li T., Liu G., He Z., Yang X. (2021). The Cd phytoextraction potential of hyperaccumulator *Sedum alfredii*-oilseed rape intercropping system under different soil types and comprehensive benefits evaluation under field conditions. Environ. Pollut..

[44] Zu Y., Qin L., Zhan F., Wu J., Li Y., Chen J., Wang J., Hu W. (2020). Effects of Intercropping of *Sonchus asper* and *Vicia faba* on plant cadmium accumulation and root responses. Pedosphere.

[45] Hei L., Wu Q.-T., Long X.-X., Hu Y.-M. (2007). Effect of co-planting of *Sedum alfredii* and *Zea mays* on Zn-contaminated sewage sludge. Huan Jing Ke Xue Huanjing Kexue.

[46] Yang M., Zhang Y., Qi L., Mei X., Liao J., Ding X., Deng W., Fan L., He X., Vivanco J.M. (2014). Plant-plant-microbe mechanisms involved in soil-borne disease suppression on a maize and pepper intercropping system. PLoS ONE.

[47] Qing L., Li-Na S., Xiao-Min H. (2015). Metabonomics study on root exudates of cadmium hyperaccumulator *Sedum alfredii*. Chin. J. Anal. Chem..

[48] Ling W., Sun R., Gao X., Xu R., Li H. (2015). Low-molecular-weight organic acids enhance desorption of polycyclic aromatic hydrocarbons from soil. Eur. J. Soil Sci..

[49] Li Y., Wang Y., Khan M.A., Luo W., Xiang Z., Xu W., Zhong B., Ma J., Ye Z., Zhu Y. (2021). Effect of plant extracts and citric acid on phytoremediation of metal-contaminated soil. Ecotoxicol. Environ. Saf..

[50] Tang X., Luo S., Huang Z., Wu H., Wang J., Shi G., He L., Xiong F., Jiang J., Liu J. (2019). Changes in the physicochemical properties and microbial communities of rhizospheric soil after cassava/peanut intercropping. bioRxiv.

[51] Tang X., Zhong R., Jiang J., He L., Huang Z., Shi G., Wu H., Liu J., Xiong F., Han Z. (2020). Cassava/peanut intercropping improves soil quality via rhizospheric microbes increased available nitrogen contents. BMC Biotechnol..

[52] Pan S., Lu R., Li H., Lin L., Li L., Xiang J., Chen L., Tang Y. (2021). Effects of mutual intercropping on cadmium accumulation in seedlings of three varieties of eggplant. Int. J. Environ. Anal. Chem..

[53] Bian F., Zhong Z., Li C., Zhang X., Gu L., Huang Z., Gai X., Huang Z. (2021). Intercropping improves heavy metal phytoremediation efficiency through changing properties of rhizosphere soil in bamboo plantation. J. Hazard. Mater..

[54] Lu Q., Li J., Chen F., Liao M.A., Lin L., Tang Y., Liang D., Xia H., Lai Y., Wang X. (2017). Effects of mutual intercropping on the cadmium accumulation in accumulator plants *Stellaria media*, *Malachium aquaticum*, and *Galium aparine*. Environ. Monit. Assess..

[55] Xia H., Liang D., Chen F., Liao M.A., Lin L., Tang Y., Lv X., Li H., Wang Z., Wang X. (2018). Effects of mutual intercropping on cadmium accumulation by the accumulator plants *Conyza canadensis*, *Cardamine hirsuta*, and *Cerastium glomeratum*. Int. J. Phytoremediation.

