# Supplementary material for: Intercropping of Euonymus japonicus with Photinia × fraseri Improves Phytoremediation Efficiency in Cd/Cu/Zn Contaminated Field
Source: Biology (Basel). 2022 Jul 28;11(8):1133. doi: 10.3390/biology11081133 (PMC9405393; doi:10.3390/biology11081133)
Supplement: Supplementary file 1 [file biology-11-01133-s001.zip › biology-1783618-supplementary.pdf]

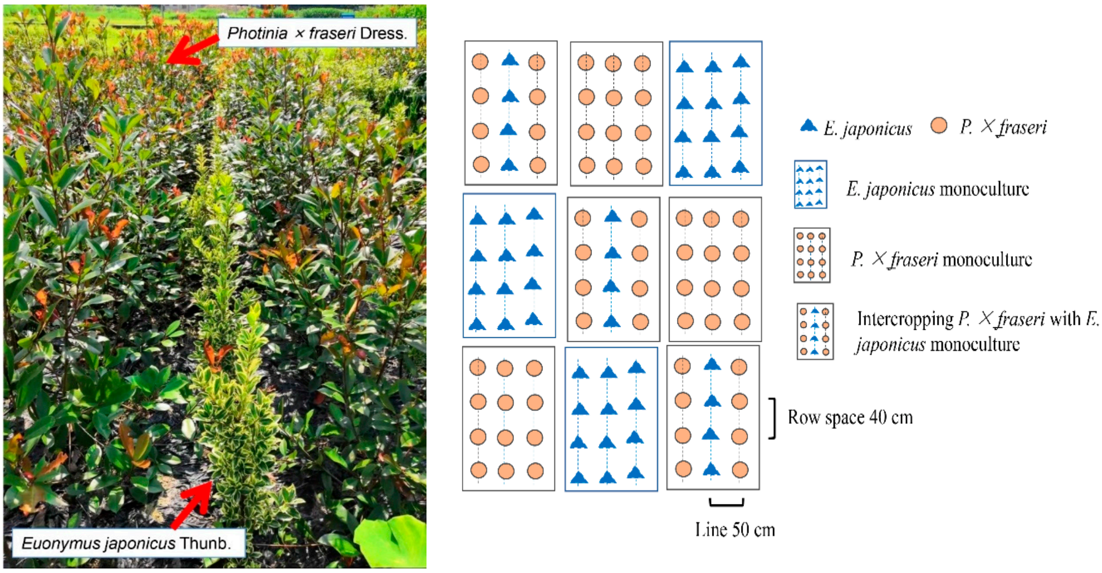

Figure.S1 Picture and schematic diagram of field experiment

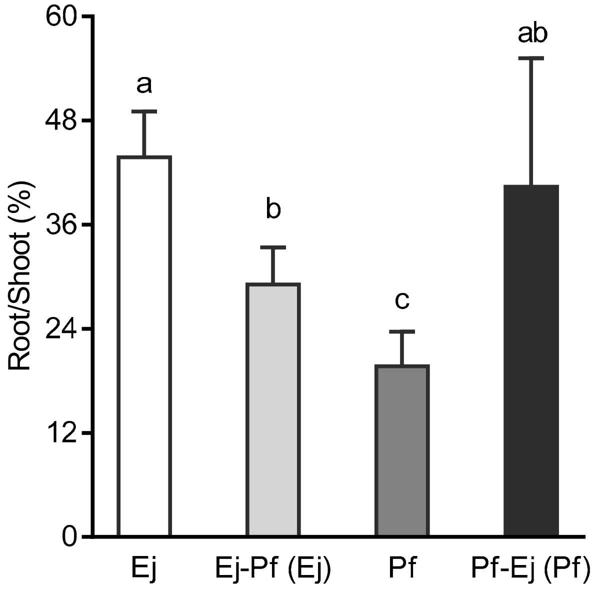

Figure.S2 The analysis of root/shoot ratio of *P.* × *fraseri* and *E. japonicus* in monoculture and intercropping systems.
